# The effects of the pandemic burden on early developmental outcomes in preterm children aged 0–3 years: a cross-sectional study

**DOI:** 10.1186/s13034-026-01112-6

**Published:** 2026-06-12

**Authors:** Miriam Cherkaoui, Volker Mall, Marcus Krüger, Verena Kraus

**Affiliations:** 1 TUM School of Medicine and Health, Department of Social Paediatrics, Technical University of Munich, Munich, Germany; 2 Division of Neonatology and Paediatric Intensive Care Medicine, Perinatal Centre, Munich Municipal Hospital Group, Munich, Germany; 3 TUM School of Medicine and Health, Department of Paediatrics, Paediatric Neurology, Technical University of Munich, Munich, Germany

**Keywords:** Developmental outcome, COVID-19 pandemic, Parenting stress, Child development, Preterm birth

## Abstract

**Background:**

The psychosocial stress experienced by families with young children during the pandemic raised concerns about developmental risks, particularly for preterm infants, who are highly sensitive to environmental influences. This study examined the association between parent-reported pandemic burden and cognitive, language and motor development of preterm children.

**Methods:**

Between 2022 and 2023, a total of 69 children (median corrected age: 21.59 months) were assessed, 62.3% of whom were born prematurely. A subset of these preterm children (*n* = 31), restricted to improve age alignment, was compared to a pre-pandemic cohort (*n* = 40; median age: 22.2 months). Development was measured using the Bayley Scales of Infant and Toddler Development (Second and Third Edition). The pandemic burden was quantified using the Corona-Index, which was based on a standardised parental questionnaire. Multiple linear regressions were conducted with the Corona-Index as the main predictor and sex, gestational age, socioeconomic status and parental stress as moderators. Group differences were analysed using Mann-Whitney-U-Tests.

**Results:**

Cognitive and motor scores did not differ significantly between cohorts, whereas language scores were significantly lower in the pandemic group (*p* = 0.001). The overall regression models for cognitive and language development did not reach statistical significance, and their sub-analyses should be interpreted with caution. Within the pandemic cohort, a significant interaction between the Corona-Index and sex was found for motor development (*p* = 0.022). Exploratory interaction effects suggested possible sex-related differences.

**Conclusion:**

Overall, preterm children assessed during the pandemic showed differences in language scores compared to the pre-pandemic control group, which may theoretically be linked to altered social environments. However, instrument-related variability and residual clinical confounding cannot be excluded. Exploratory analyses highlighted the importance of examining sex-specific patterns in the context of environmental disruptions.

**Supplementary Information:**

The online version contains supplementary material available at 10.1186/s13034-026-01112-6.

## Introduction

Neurological immaturity underlies the increased vulnerability of infants born preterm. Disruptions of late gestational brain development, particularly affecting cortical growth and white matter organization, increase the risk for impairments in cognitive, language, motor, and sensory domains. Preterm children have approximately twice the risk of neurodevelopmental disability compared to term-born peers, with cognitive deficits being the most common outcome [1]. Within the preterm populations, boys have been reported to be at greater risk for adverse neurodevelopmental outcomes compared to girls [1, 14]. Diverse organic neonatal conditions and environmental factors may further exacerbate these vulnerabilities and are associated with poorer developmental outcomes in early childhood [2, 3]. The COVID-19 pandemic is an important environmental factor of recent years. It led to contact restrictions, which have substantially altered the psychosocial environment in which early child development takes place. Mask wearing and increased screen time limited social interaction with humans, which is a crucial factor for early language and social learning [8, 13]. Beyond social interactions of the child with the environment the psychosocial environment in the core family itself was altered. Social isolation reduced access to healthcare and support services, and disruptions to daily routines were the most prominent environmental alterations [8, 13]. These multidimensional alterations imposed by the pandemic are known as the pandemic burden. Pandemic burden is intertwined with the stress experienced by individuals and families. Studies conducted during the pandemic consistently report elevated levels of parental stress [7], including increased symptoms of distress, depression, and anxiety among caregivers of young children [4]. Higher parental stress has been associated with less optimal parent–child interactions and, in turn, with adverse developmental outcomes, particularly in behavioural and socio-emotional domains [10–12]. These domains were reflected by higher rates of regulatory disorders, including increased crying, feeding difficulties, and sleep disturbances compared to pre-pandemic studies [5, 6], . These regulatory problems have in turn been linked to elevated parental stress levels. This loop suggests a potential pathway through which pandemic-related conditions may in turn affect early development [7].

Despite the growing literature on preterm development and the psychosocial consequences of the pandemic, several important gaps remain. Existing studies on preterm children have predominantly focused on medical and biological risk factors [2, 3, 15, 16], whereas psychosocial influences, particularly pandemic-specific parental stress, have received less attention. At the same time, studies examining early child development during the COVID-19 pandemic have predominantly focused on term-born populations, leaving the vulnerable population of preterm children underrepresented [5, 8]. Moreover, previous studies have often relied on broad indicators of family stress rather than distinguishing pandemic-specific stressors [10–12]. Finally, although sex differences in early development are well documented, their role in shaping vulnerability to the pandemic burden in preterm children has not been systematically examined. Taken together, these gaps point to the need for a more integrated analysis of biological vulnerability and pandemic-related psychosocial risk with a focus on preterm infants.

The present study addresses these gaps by examining developmental outcomes in preterm children aged 0–3 years during the COVID-19 pandemic and by comparing a pandemic cohort with a pre-pandemic cohort of preterm children. Within the pandemic cohort, we investigate whether pandemic-specific parental stress is associated with children’s cognitive, language, and motor development, and whether these associations differ by sex.

We hypothesized that preterm children assessed during the pandemic would show lower standardized Bayley composite scores in developmental testing compared to the pre-pandemic preterm cohort. Furthermore, we expected higher levels of pandemic-specific parental stress to be associated with poorer cognitive, language, and motor outcomes, with stronger adverse effects in boys than in girls.

## Methods

### Participants

The pandemic cohort consisted of 69 children aged 6 to 42 months (M = 22.36; IQR = 14.53–26.66 months), assessed between May 2022 and June 2023. 31 of those children are later described as the pandemic group of preterms. Participants were recruited from outpatient follow-up care at the Department of Neonatology of the Munich Municipal Hospital and from inpatient services at the Social Pediatric Center (kbo-Kinderzentrum München Großhadern). Because these recruitment settings may differ systematically regarding clinical severity and developmental risk, a potential selection bias cannot be excluded. Parents provided written informed consent prior to participation. Ethical approval was obtained from the Ethics Committee of the Technical University of Munich. The inclusion criterion was an age between 6 and 42 months, meaning that all children were born either during the pandemic or shortly before it. Complete demographic data on the participants can be found in Table 1.

**Table 1:** Demographic data of the pandemic group (*n* = 69) devided by sex

	Pandemic group females (*n* = 41)	Pandemic group males (*n* = 28)
Preterm birth < 37 weeks (%)	56.1	71.4
Corrected age in months: median (IQR)	23.18 (13.09–26.04)	22.73 (12.42–25.12)
Age in months: median (IQR)	25.51 (15.08–28.35)	24.32 (14.01–26.67)
Gestational age in weeks: median (IQR)	33.0 (29.4–38.3)	34.0 (31.9–37.1)
Birthweight in grams: median (IQR)	1500 (990–2543)	1930 (1400–2850)
Multiple gestation (%)	22.0	21.4

The sample included 43 children who were born preterm (< 37 weeks of gestation). To examine the potential impact of the pandemic on the early childhood development in this vulnerable population, preterm children from the pandemic cohort were compared to a pre-pandemic cohort of preterm infants (Table 2). In the original pandemic preterm subgroup (*n* = 43), gestational age and birthweight differed substantially from those of the pre-pandemic cohort. Given the strong influence of these variables on developmental outcomes, a restriction procedure was applied to reduce heterogeneity and improve comparability between cohorts. Specifically, 12 children at the extremes of the age distribution were excluded symmetrically from both ends, resulting in an analytic pandemic preterm subgroup of 31 children. This procedure was implemented after baseline imbalances between cohorts had been observed and therefore represents a post hoc analytic modification. Consequently, the cohort comparisons should be interpreted with caution and considered exploratory in nature. Although the restriction procedure improved comparability for some variables, residual confounding remains likely, particularly given the remaining differences in gestational age and birthweight distributions between cohorts. Accordingly, observed differences may reflect underlying clinical heterogeneity rather than effects of pandemic exposure. Furthermore, this data-driven restriction procedure may have introduced selection bias and may limit the generalizability of the findings. For transparency, we have included the descriptive statistics of the original, unrestricted pandemic preterm subgroup (*n* = 43), enabling direct comparison of how the restriction affected sample characteristics.

The pre-pandemic cohort consisted of 40 formerly preterm children and corresponds to the “Phase − 1” group of the Munich cohort described in Lau & Kraus, 2023, assessed between 2018 and 2020 prior to the first COVID-19 lockdown. Children were recruited and assessed as outpatients in the Neonatology Department at Munich Municipal Hospital, which represents the primary recruitment pathway also used for the pandemic cohort. As this study involved retrospective data analysis, informed consent was not necessary for the pre-pandemic study group. The cohort comprised all infants who underwent clinical developmental testing during this period. However, differences in recruitment procedures, referral patterns, and inclusion criteria cannot be fully excluded and may affect comparability. In this cohort, 45% of the children were female and 22.5% were multiples. The median gestational age at birth was 30.1 (27.1–31.3) weeks and the median birth weight was 1220.0 (937.5–1477.5) grams [17]. Complete demographic data on the participants can be found in Table 2.

**Table 2:** Demographic data of the preterm cohorts

	Pre-pandemic group of preterms (*n* = 40)	Unrestricted pandemic group of preterms (*n* = 43)	Restricted pandemic group of preterms (*n* = 31)
Male sex (%)	55	46.5	45.2
Corrected age in months: median (IQR)	22.2 (22.0–23.1)	22.92 (12.38–24.38)	23.82 (22.06–24.81)
Age in months: median (IQR)	24.85 (24.28–25.48)	24.69 (14.87–26.55)	26.20 (23.33–26.73)
Gestational age in weeks: median (IQR)	30.1 (27.1–31.3)	33.0 (29.6–34.2)	31.1 (29.4–34.0)
Very preterm < 32 weeks (%)	82.5	44.2	51.6
Birthweight in grams: median (IQR)	1220.0 (937.5–1477.5)	1470.0 (1023.5–2000.0)	1415 (940.0–1850.0)
Very low birthweight < 1500 g (%)	87.5	52.8	61.5
Multiple gestation (%)	22.5	34.9	35.5

### Survey instruments

The child’s cognitive, motor and language development were assessed using the Bayley Scales of Infant and Toddler Development, Third Edition (Bayley-III). The Bayley Scales of Infant Development, Second and Third Edition (BSID-II and Bayley-III), are well-established instruments for the assessment of early developmental outcomes and have demonstrated good reliability and validity in both general and preterm populations. Previous research has shown that the Bayley-III provides reliable and valid estimates of cognitive, language, and motor development in preterm infants, although some studies suggest a tendency to yield slightly higher scores compared to earlier versions of the scale. Overall, the instruments are considered appropriate for assessing developmental outcomes in this population [18]. The pandemic burden was assessed using a standardized questionnaire. The Corona-Index was derived from 32 items covering multiple domains of the pandemic burden, including social isolation, reduced support services, caregiving challenges, family conflict, financial strain, work-related changes, infection-related concerns, and perceived burden associated with the pandemic. The Items were rated on ordinal Likert-type scales. For the present study, the relevant items were summed to create a total burden score and subsequently divided by 5 to derive the Corona-Index, with higher values indicating greater pandemic burden. The index captures multiple dimensions of the pandemic burden. Therefore, internal consistency is moderate (Cronbach’s α = 0.66). The questionnaire is based on instruments used in the CoronabaBY study and has been applied in previous research on pandemic burden in families with young children [9, 19]. In these studies, perceived pandemic burden showed small to moderate correlations with established measures of parental stress, such as the Parenting Stress Index (Eltern-Belastungsinventar, EBI), supporting its convergent validity (e.g., ρ ≈ 0.27). While the Corona-Index represents a standardized measure capturing multiple domains of pandemic burden, it has not been independently validated as a psychometric instrument. This should be considered when interpreting the results. Sociodemographic data, including parental education, occupation, family structure and socio-economic status was obtained using a standardized demographic questionnaire. Based on the occupational information provided, the Nam-Powers-Boyd-Index was calculated as an indicator of socioeconomic status. The index assigns a socioeconomic score to each occupation. It is based on the median education and median income of individuals within that occupation in large population datasets. Occupations are ranked separately according to median educational attainment and median earnings. These rankings are combined into a composite percentile score ranging from 0 to 100. The score reflects the relative socioeconomic position of each occupation. Higher values indicate occupations with higher average levels of education and income. The score assigned to an individual therefore represents the typical socioeconomic standing associated with their reported occupation rather than individual-level characteristics [20]. In addition, parental psychological stress was assessed using the Symptom Checklist-90 (SCL-90), from which the Global Severity Index (GSI) was derived as a summary measure of overall psychological distress [21]. The GSI provides a comprehensive indicator of general psychological distress across multiple symptom dimensions. It is widely used in research as a global measure of mental health and is particularly suitable for capture overall parental stress in the context of the pandemic [21]. The pre-pandemic cohort of preterm children was assessed using the Bayley Scales of Infant Development, Second Edition (BSID-II) [22].

### Procedure and data analysis

The Bayley Scales of Infant and Toddler Development III administered in person by trained examiners. Assessments were conducted under controlled testing conditions, including a structured testing environment and standardized instructions. Raw scores were recorded during the assessment and subsequently converted into composite scores (cognitive, language, and motor) [23].

One parent completed the questionnaires regarding demographic information, pandemic-specific stressors and parental psychological distress.

The children in the pre-pandemic cohort were assessed using the Bayley Scales of Infant Development, Second Edition (BSID-II), which was administered in person by the same trained social pedagogue [17].

Analyses of the Bayley-III data were performed with IBM SPSS Statistics Version 29.0.1.1. Raw scores were converted into composite scores (Cognitive Composite Score, Motor Composite Score, and Language Composite Score) according to the test manual’s scoring procedure [23].

Since different Bayley Scale versions were used, scale comparability was carefully considered. In the study comparing Bayley-II and -III values in a randomised controlled trial, children reached higher scores in Bayley-III than in Bayley-II testing. The mean difference for the Mental Developmental Index (MDI) was calculated as + 14.1 ± 12.9 for the Cognitive Scale and + 9.8 ± 11.8 for the Language Scale. The mean difference for the Psychomotor Developmental Index (PDI) was calculated at + 9.0 ± 11.9 for the Motor Scale. Based on published mean differences, Bayley-II scores were adjusted by + 14.1 points for cognition, + 9.8 points for language, and + 9.0 points for motor development to improve comparability across cohorts [17, 24]. This adjustment represents a pragmatic approach and cannot fully account for variability between the two test versions. The use of fixed mean adjustments to align Bayley-II and Bayley-III scores represents a methodological limitation. Given the large variability reported in the literature, this approach assumes a level of comparability that may not be justified at the individual level and may introduce measurement bias. Importantly, we cannot exclude that part of the observed group differences reflects residual inconsistencies between assessment instruments rather than true developmental differences. Therefore, findings involving cross-cohort comparisons should be interpreted with particular caution. Prior to statistical analysis, data distributions were examined for normality. Since the Bayley III Composite Scores from the pandemic group were not normally distributed, Mann-Whitney-U-Tests were used to compare the two groups. For transparency, additional Mann–Whitney U analyses using the unadjusted Bayley-II scores as well as the upper and lower bound adjustments are provided in the Supplementary Material (Table A1).

A series of multiple linear regression analysis were conducted to examine the relationship between pandemic burden and the development of children in the pandemic cohort (*n* = 69). The Cognitive, Language, and Motor Composite Scores were used as the dependent variables and the Corona-Index as the main predictor. Each model included four moderator variables (sex of the child, gestational age, socio-economic status measured by the Nam-Powers-Boyd-Index and parental psychological stress measured by the Global Severity Index) as well as interaction terms between the Corona-Index and the sex of the child, to examine potential moderating effects on developmental outcomes. The statistical significance was set at a level of *p* ≤ 0.05. To examine whether age at assessment influenced the results, additional regression analyses were conducted including age as a covariate. While the inclusion of age led to minor changes in some coefficients and their statistical significance, the overall pattern of findings remained stable. In particular, the direction of the main effects and the general interpretation of the models were largely unchanged. Detailed results of these analyses are provided in the supplementary material (Tables A2, A3, A4).

While non-parametric tests were used for group comparisons due to non-normality of the outcome variables, multiple linear regression was applied to model associations within the pandemic cohort, as linear regression is generally robust to moderate deviations from normality, particularly when focusing on estimates of associations rather than distributional assumptions of the dependent variable. Model assumptions were evaluated by inspection of residuals, which did not indicate substantial violations of linearity or homoscedasticity [25].

Given the relatively small sample size, the number of predictors included in the models was deliberately restricted to avoid overfitting. Covariates were selected based on a combination of theoretical relevance and empirical evidence from previous research on early child development. Specifically, variables known to be associated with developmental outcomes in preterm populations were included as core predictors. Interaction terms were limited to a small number of pre-specified hypotheses, in particular the interaction between sex and the Corona-Index, as sex-specific vulnerability patterns in early development have been reported in the literature. No additional exploratory interaction terms were included in order to maintain model parsimony. Despite these precautions, the inclusion of multiple predictors and interaction terms in a relatively small sample may still increase the risk of overfitting. Therefore, results should be interpreted cautiously and considered exploratory. In addition, the inclusion of multiple regression models and interaction analyses increases the risk of Type I error, particularly given the relatively small complete-case sample and exploratory nature of the analyses.

Missing data were handled using a complete-case approach. The proportion of missing data varied across variables. While no missing values were observed for gestational age, sex, or Corona-Index, missingness was present for the Nam-Powers-Boyd Index (29%) and the Global Severity Index (23%). As a result, regression analyses were based on 45 complete cases out of the total sample of 69 participants (34.8% case-wise exclusion). Given the relatively high proportion of excluded cases, complete-case analysis may have reduced statistical stability and introduced selection bias if participants with missing data systematically differed from those included in the analyses. Consequently, the regression findings should be interpreted with caution, particularly given the relatively small final analytical sample size and the exploratory rather than confirmatory nature of the models.

## Results

**Fig. 1:**
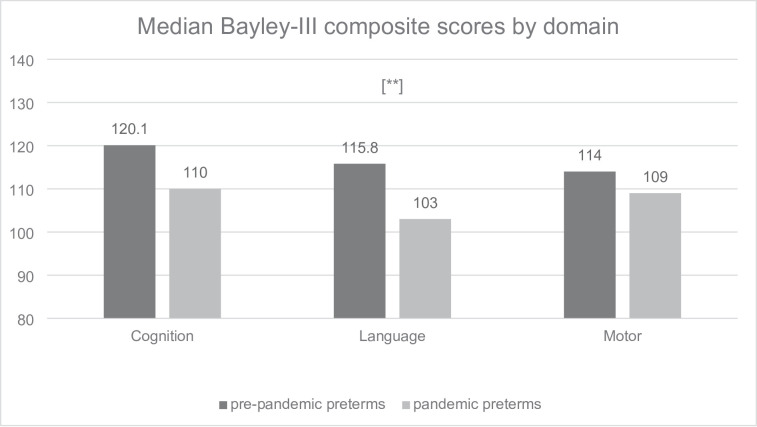
Bayley-III composite scores by domain. * statistically significant at the 5% level (*p* < 0.05). ** statistically significant at the 1% level (*p* < 0.01). *** statistically significant at the 0.1% level (*p* < 0.001)

The Mann-Whitney-U-Test showed a statistically significant difference in the Language Composite Scores between cohorts. The pre-pandemic group had a median score of 115.8 (IQR: 108.8–119.8), whereas the pandemic cohort showed a lower median score of 103.0 (IQR: 85.5–114). (Table 3; Fig. 1). The Mann-Whitney-U-Test revealed a statistically significant group difference (U = 1212.0, *p* = 0.001). According to the Bayley-III classification, composite scores are standardized to a mean of 100 with a standard deviation of 15, and scores between 85 and 115 are typically considered within the normative range. Thus, although the pandemic cohort showed lower median scores, the central tendency of both groups remained within the expected normative range, indicating that the observed difference, while statistically significant, should be interpreted with caution regarding its clinical relevance.

There were no significant differences in the results of cognitive and motor development between the two groups. A Mann-Whitney-U-Test yielded no statistically significant difference. These results suggest that there is no evidence of statistically significant differences between cohorts in these domains based on the present data (Table 3; Fig. 1).

**Table 3:** Standardized Bayley composite scores by domain

Domain	Pre-pandemic preterms (*n* = 40)	Pandemic preterms (*n* = 31)	*p*-value
Cognitive composite score: median (IQR)	120.1 (113.1–124.1)	110.0 (97.5–125.0)	0.078
Language composite score: median (IQR)	115.8 (108.8–119.8)	103.0 (85.5–114.0)	0.001**
Motor composite score: median (IQR)	114.0 (110.0–122.5)	109.0 (100.0–119.0)	0.105

*statistically significant at the 5% level (*p* < 0.05)

**statistically significant at the 1% level (*p* < 0.01)

***statistically significant at the 0.1% level (*p* < 0.001)

Multiple linear regression analysis including an exploratory interaction term between Corona-Index and sex (coded as male = 1, female = 0; female as the reference category) was performed to examine their association with cognitive development. The overall regression model was not statistically significant (*p* = 0.302, R² = 0.17, adjusted R² = 0.03). Within this model, the interaction between the Corona-Index and sex reached nominal statistical significance (B = 4.12, t(45) = 2.173, *p* = 0.036). However, significant predictors within non-significant overall models should be interpreted cautiously. The exploratory interaction term suggests possible sex-related differences regarding the association between pandemic burden and cognitive outcomes, but the direction and magnitude of this relationship cannot be interpreted reliably based on the present data. No definitive conclusions regarding sex-specific effects can therefore be drawn from this model (Table 4).

A multiple linear regression analysis with an exploratory interaction term between the Corona-Index and sex was performed to examine the influence of these two factors on the language development. The overall model did not reach statistical significance (*p* = 0.175, R² = 0.20, adjusted R² = 0.08), and the interaction between the Corona-Index and sex was also not significant (B = 3.71, t(45) = 1.710, *p* = 0.095). A significant main effect of sex was observed (B = -33.19, t(45) = -2.112, *p* = 0.041), indicating differences in language scores between boys and girls within the model. However, significant predictors within non-significant overall models should be interpreted cautiously. No evidence was found for an interaction between pandemic burden and sex in relation to language outcomes (Table 5).

A multiple linear regression analysis including an exploratory interaction term between Corona-Index and sex was performed to examine the influence on the motor development. The overall regression model was statistically significant (*p* = 0.019, R² = 0.32, adjusted R² = 0.21). Exploratory interaction effects within this model suggested possible sex-related differences regarding the association between pandemic burden and motor outcomes (B = 3.95, t(45) = 2.380, *p* = 0.022). In addition, sex showed a significant main effect within the model (*p* = 0.013) (Table 6).

**Table 4:** Multiple linear regression model with interaction between Corona-Index and sex to examine the influence on the cognitive composite score (*n* = 45)

	Regression coefficient B	Standard error	t-value	*p*-value	95.0% Confidence interval for B		
					Lower bound	Upper bound	
(Intercept)	129.604	29.513	4.391	< 0.001	69.858	189.351	
Corona-Index	-0.420	1.123	-0.374	0.711	-2.693	1.854	
Sex	-32.740	13.740	-2.383	0.022*	-60.556	-4.925	
Nam-Powers-Boyd-Index	-0.070	0.135	-0.518	0.607	-0.344	0.204	
Gestational age	-0.411	0.795	-0.518	0.608	-2.020	1.198	
Global-Severity-Index	0.043	0.086	0.493	0.625	-0.132	0.217	
Corona-Index: Sex	4.119	1.896	2.173	0.036*	0.282	7.957	

*statistically significant at the 5% level (*p* < 0.05)

**Table 5:** Multiple linear regression model with interaction between Corona-Index and sex to examine the influence on the language composite score (*n* = 45)

	Regression coefficient B	Standard error	t-value	*p*-value	95.0% Confidence interval for B	
					Lower bound	Upper bound
(Intercept)	87.860	33.752	2.603	0.013	19.532	156.187
Corona-Index	0.420	1.284	0.327	0.746	-2.181	3.020
Sex	-33.194	15.713	-2.112	0.041*	-65.004	-1.383
Nam-Powers-Boyd-Index	0.217	0.155	1.406	0.168	-0.096	0.531
Gestational age	-0.148	0.909	-0.163	0.872	-1.988	1.692
Global-Severity-Index	0.007	0.099	0.069	0.945	-0.193	0.207
Corona-Index: Sex	3.706	2.168	1.710	0.095	-0.682	8.095

* statistically significant at the 5% level (*p* < 0.05)

**Table 6:** Multiple linear regression model with interaction between Corona-Index and sex to examine the influence on the motor composite score (*n* = 45)

	Regression coefficient B	Standard error	t-value	*p*-value	95.0% Confidence interval for B	
					Lower bound	Upper bound
(Intercept)	129.833	25.835	5.025	< 0.001	77.532	182.133
Corona-Index	-0.028	0.983	-0.029	0.977	-2.018	1.962
Sex	-31.525	12.028	-2.621	0.013*	-55.874	-7.176
Nam-Powers-Boyd-Index	0.223	0.118	1.884	0.067	-0.017	0.463
Gestational age	-1.236	0.696	-1.776	0.084	-2.644	0.173
Global-Severity-Index	0.104	0.076	1.380	0.176	-0.049	0.257
Corona-Index: Sex	3.950	1.659	2.380	0.022*	0.591	7.309

*statistically significant at the 5% level (*p* < 0.05)

Descriptive analyses stratified by sex showed small differences in developmental scores between boys and girls across all domains (Table 7). Boys had slightly lower mean scores in cognitive development (105 vs. 106), language development (93 vs. 98), and motor development (103 vs. 106). In addition, boys showed higher mean values on the Corona-Index (6.6 vs. 3.8), indicating higher reported levels of pandemic burden in this group. Overall, these descriptive findings indicate minor differences between boys and girls, however, the magnitude of these differences is small, and no formal statistical tests were conducted. Therefore, these patterns should be interpreted cautiously and not as evidence of systematic sex differences.

**Table 7:** Descriptive comparison of sex-specific mean values

Variable	Girls (Mean)	Boys (Mean)
Cognitive composite score	106 ± 18	105 ± 23
Language composite score	98 ± 17	93 ± 29
Motor composite score	106 ± 18	103 ± 20
Corona-index	3.8 ± 3.9	6.6 ± 3.2

## Discussion

The present study examined the association between pandemic burden and early developmental outcomes in preterm children. Overall, the results do not support the hypothesis of a general negative impact of the COVID-19 pandemic on developmental outcomes. A statistically significant difference was observed for language composite scores, which were lower in the pandemic cohort compared to the pre-pandemic cohort, while no statistically significant group differences were found for cognitive and motor outcomes. Importantly, all developmental scores remained within the normative range. Previous research has suggested that early language development may be particularly sensitive to environmental and social contexts [3, 26]. During the pandemic period, changes in childcare attendance, peer interaction, daily routines, and family environments were widely reported in many populations. In this context, some children may have experienced reduced diversity of social and linguistic input. Prior studies have reported associations between reduced access to visual communicative cues, including facial expressions during mask-wearing, and lower expressive vocabulary scores in young children [19, 27]. However, these contextual factors were not directly measured in the present study. Consequently, these considerations should be viewed as theoretical possibilities, informed by previous research, rather than as empirically demonstrated pathways within the current dataset.

No statistically significant group differences were found for cognitive or motor outcomes. Although previous research studies have suggested that motor development is more strongly associated with biological maturation, potentially being less dependent on external social input and more resilient to contextual disruptions [3, 15, 28] the absence of statistically significant differences between groups should not be interpreted as evidence of equivalence.

The analyses further explored potential sex-related differences in the association between pandemic burden and developmental outcomes. While interaction effects between Corona-Index and sex were observed in some models, these findings were inconsistent across developmental domains and partly emerged in models that were not statistically significant overall. Given the relatively small complete-case sample, multiple predictors, and exploratory nature of the analyses, these findings should be interpreted cautiously and regarded as exploratory rather than confirmatory.

Several methodological limitations should be considered. Due to the retrospective design inherent to the research question, developmental assessments from different time periods had to be compared across the Bayley-II and Bayley-III editions. The comparability of the Bayley-II and Bayley-III instruments remains limited despite the applied score adjustment procedure. Although previously reported mean differences between the scales were used to adjust scores, this approach assumes a constant shift between instruments. However, the large standard deviations reported in the literature indicate substantial variability in these differences, suggesting that the relationship between Bayley-II and Bayley-III scores is not uniform across individuals. Consequently, a simple mean-based adjustment is unlikely to fully account for this variability and may introduce measurement error or bias into the estimated developmental outcomes [24]. Importantly, we cannot exclude that part of the observed differences between cohorts reflects residual inconsistencies between assessment instruments rather than true developmental differences.

The age-based restriction procedure that was applied to improve comparability between cohorts after baseline imbalances had been observed. This data-driven, post hoc restriction procedure may have introduced selection bias and may limit the generalizability of the findings. Although the procedure improved comparability for some variables, relevant differences in gestational age and birthweight distributions remained between cohorts. Therefore, residual confounding remains likely despite the restriction procedure, and the cohort comparisons should be interpreted as exploratory rather than confirmatory.

Furthermore, the relatively small sample size and complete-case analysis with substantial case-wise exclusion may have reduced statistical stability and introduced selection bias, particularly in the regression analyses. In addition, the inclusion of multiple regression models and interaction analyses increases the risk of Type I error.

In addition, the Corona-Index represents a study-specific composite measure with limited independent validation. Potential inter-rater variability and differences in recruitment settings between cohorts may also have influenced the findings. Finally, the cross-sectional observational design precludes causal conclusions and does not allow conclusions about developmental trajectories over time.

## Conclusion

This study indicates potential differences in language development between preterm children assessed before and during the COVID-19 pandemic, while no statistically significant differences were observed for cognitive and motor outcomes. All developmental scores remained within the normative range.

Given the observational design and methodological limitations, these findings should be interpreted as exploratory associations observed during the COVID-19 pandemic rather than as evidence of causal developmental effects. The results underscore the complexity of early developmental trajectories and suggest that potential effects of pandemic-related circumstances may vary across developmental domains and are likely influenced by a combination of societal, familial, and individual factors.

## Supplementary Information

Below is the link to the electronic supplementary material.

Supplementary Material 1.

## Data Availability

The datasets supporting the conclusions of this article are included within the article itself. Any further data used and analysed during the current study are available from the corresponding author upon reasonable request.

## Abbrevations

BSID-II: Bayley scales of infant development.bayley scales of infant development, second edition
Bayley-III: Bayley scales of infant and toddler development, third edition
MDI: Mental development index
PDI: Psychomotor development index
SCL: Symptom checklist
GSI: Global severity index
NPB: Nam-Powers-Boyd-Index
VLBW: Very low birth weight
VPT: Very preterm

## Declarations

TUM School of Medicine and Health (vote no. 2022-224-S-NP).
