# Supplementary material for: The effects of the pandemic burden on early developmental outcomes in preterm children aged 0–3 years: a cross-sectional study
Source: Child Adolesc Psychiatry Ment Health. 2026 Jun 12;20:97. doi: 10.1186/s13034-026-01112-6 (PMC13386862; doi:10.1186/s13034-026-01112-6)
Supplement: Supplementary file 1 — Supplementary Material 1. [file 13034_2026_1112_MOESM1_ESM.docx]

Appendix

Table A1 Sensitivity Analysis of Bayley-II Adjustment Procedures and Corresponding Mann–Whitney U Test Results for Cross-Cohort Comparisons

| **Domain** | **Pre-pandemic preterms (n=40)**  **Lower Bound Correction** | **Pre-pandemic preterms (n=40)**  **Upper Bound Correction** | **Pre-pandemic preterms (n=40)**  **No Correction** | | **Pre-pandemic preterms (n=40)**  **Fixed Mean Correction** | **Pandemic preterms (n=31)** |
| --- | --- | --- | --- | --- | --- | --- |
| Cognitive composite score: median (IQR) | 107.2 (100.2 – 111.2) | 133.0 (126.0 – 137.0) | 106.0 (99.0 – 110.0) | | 120.1 (113.1 – 124.1) | 110.0 (97.5 – 125.0) |
| p-value of the Mann-Whitney-U test | 0.296 | **<0.001**** | 0.172 | | 0.078 |  |
| Language composite score: median (IQR) | 104.0 (97.0 – 108.0) | 127.6 (120.6 – 131.6) | | 106.0 (99.0 – 110.0) | 115.8 (108.8 – 119.8) | 103.0 (85.5 – 114.0) |
| p-value of the Mann-Whitney-U test | *0.535* | ***<0.001***** | | *0.248* | **0.001**** |  |
| Motor composite  score: median (IQR) | 102.1 (98.1 – 110.6) | 125.9 (121.9 – 134.4) | | 105.0 (101.0 – 113.5) | 114.0 (110.0 – 122.5) | 109.0 (100.0 – 119.0) |
| p-value of the Mann-Whitney-U test | **0.024*** | **<0.001**** | | 0.282 | 0.105 |  |

Table A2 Multiple linear regression model for the cognitive scale value including age as a covariate

| **Coefficients^a^** | | | | | | | | | | |
| --- | --- | --- | --- | --- | --- | --- | --- | --- | --- | --- |
| Model | | Unstandardized Coefficients | | Standardized Coefficients | t-value | p-value | 95.0% Confidence Interval for B | | Collinearity Statistics | |
|  |  | Regression Coefficient B | Std.-Error | Beta |  |  | Lower Bound | Upper Bound | Tolerance | VIF |
| 1 | (Intercept) | 116.825 | 14.563 |  | 8.022 | <0.001 | 87.318 | 146.331 |  |  |
|  | Age | -0.419 | 0.357 | -0.189 | -1.175 | 0.248 | -1.143 | 0.304 | 0.800 | 1.250 |
|  | Corona-Index | -0.006 | 1.104 | -0.001 | -0.005 | 0.996 | -2.242 | 2.231 | 0.583 | 1.716 |
|  | Sex | -32.777 | 13.345 | -0.866 | -2.456 | 0.019 | -59.817 | -5.737 | 0.166 | 6.032 |
|  | Nam-Powers-Boyd-Index | -0.136 | 0.135 | -0.150 | -1.004 | 0.322 | -0.410 | 0.138 | 0.927 | 1.079 |
|  | Gestational age | 13.135 | 7.499 | 0.337 | 1.752 | 0.088 | -2.060 | 28.330 | 0.556 | 1.799 |
|  | Global-Severity-Index | 0.114 | 0.085 | 0.251 | 1.339 | 0.189 | -0.059 | 0.287 | 0.588 | 1.701 |
|  | Corona-Index:Sex | 3.760 | 1.846 | 0.786 | 2.036 | 0.049 | 0.019 | 7.501 | 0.138 | 7.224 |
| a. Dependent Variable: Cognitive Composite Score | | | | | | | | | | |

Table A3 Multiple linear regression model for the language scale value including age as a covariate

| **Coefficients^a^** | | | | | | | | | | |
| --- | --- | --- | --- | --- | --- | --- | --- | --- | --- | --- |
| Model | | Unstandardized Coefficients | | Standardized Coefficients | t-value | p-value | 95.0% Confidence Interval for B | | Collinearity Statistics | |
|  |  | Regression Coefficient B | Std.-Error | Beta |  |  | Lower Bound | Upper Bound | Tolerance | VIF |
| 1 | (Intercept) | 82.940 | 16.348 |  | 5.074 | <0.001 | 49.817 | 116.064 |  |  |
|  | Age | -0.497 | 0.401 | -0.191 | -1.240 | 0.223 | -1.309 | 0.315 | 0.800 | 1.250 |
|  | Corona-Index | 0.794 | 1.239 | 0.116 | 0.641 | 0.526 | -1.717 | 3.305 | 0.583 | 1.716 |
|  | Sex | -33.182 | 14.981 | -0.750 | -2.215 | 0.033 | -63.536 | -2.827 | 0.166 | 6.032 |
|  | Nam-Powers-Boyd-Index | 0.134 | 0.152 | 0.127 | 0.883 | 0.383 | -0.174 | 0.442 | 0.927 | 1.079 |
|  | Gestational age | 17.554 | 8.419 | 0.386 | 2.085 | 0.044 | 0.497 | 34.612 | 0.556 | 1.799 |
|  | Global-Severity-Index | 0.126 | 0.096 | 0.237 | 1.320 | 0.195 | -0.068 | 0.320 | 0.588 | 1.701 |
|  | Corona-Index:Sex | 3.274 | 2.073 | 0.586 | 1.580 | 0.123 | -0.925 | 7.473 | 0.138 | 7.224 |
| a. Dependent Variable: Language Composite Score | | | | | | | | | | |

Table A4 Multiple linear regression model for the motor scale value including age as a covariate

| **Coefficients^a^** | | | | | | | | | | | |
| --- | --- | --- | --- | --- | --- | --- | --- | --- | --- | --- | --- |
| Model | | Unstandardized Coefficients | | Standardized Coefficients | t-value | p-value | 95.0% Confidence Interval for B | | Collinearity Statistics | |  |
|  |  | Regression Coefficient B | Std.-Error | Beta |  |  | Lower Bound | Upper Bound | Tolerance | VIF |  |
| 1 | (Intercept) | 78.132 | 12.524 |  | 6.238 | <0.001 | 52.755 | 103.508 |  |  |  |
|  | Age | -0.007 | 0.307 | -0.003 | -0.022 | 0.983 | -0.629 | 0.615 | 0.800 | 1.250 |  |
|  | Corona-Index | 0.256 | 0.949 | 0.045 | 0.270 | 0.789 | -1.667 | 2.180 | 0.583 | 1.716 |  |
|  | Sex | -29.992 | 11.477 | -0.819 | -2.613 | 0.013 | -53.247 | -6.738 | 0.166 | 6.032 |  |
|  | Nam-Powers-Boyd-Index | 0.163 | 0.116 | 0.185 | 1.397 | 0.171 | -0.073 | 0.399 | 0.927 | 1.079 |  |
|  | Gestational age | 18.321 | 6.450 | 0.486 | 2.841 | 0.007 | 5.252 | 31.389 | 0.556 | 1.799 |  |
|  | Global-Severity-Index | 0.144 | 0.073 | 0.328 | 1.969 | 0.056 | -0.004 | 0.293 | 0.588 | 1.701 |  |
|  | Corona-Index:Sex | 3.214 | 1.588 | 0.694 | 2.024 | 0.050 | -0.004 | 6.431 | 0.138 | 7.224 |  |
| a. Dependent Variable: Motor Composite Score | | | | | | | | | | | |
